# Evaluation of ATP bioluminescence for rapid determination of cleanliness of livestock trailers after a commercial wash

**DOI:** 10.1093/tas/txae052

**Published:** 2024-04-05

**Authors:** Flavia G Letsch, Michael W Welch, Matthew Meyer, Grant A Hedblom, Eric Parr, Dyneah M Classen, Molly Dillard, Dustin D Boler

**Affiliations:** Carthage Veterinary Service Ltd., Carthage, IL 62321, USA; Carthage Veterinary Service Ltd., Carthage, IL 62321, USA; Neogen Corporation, Lansing, MI 48912, USA; Neogen Corporation, Lansing, MI 48912, USA; Carthage Veterinary Service Ltd., Carthage, IL 62321, USA; Carthage Veterinary Service Ltd., Carthage, IL 62321, USA; Carthage Veterinary Service Ltd., Carthage, IL 62321, USA; Carthage Veterinary Service Ltd., Carthage, IL 62321, USA

**Keywords:** ATP bioluminescence, biosecurity, cleanliness, luminometer, trailer wash

## Abstract

Pathogens such as porcine epidemic diarrhea virus (PEDV), porcine reproductive and respiratory syndrome (PRRSV), and *E. coli* are known to spread by contaminated vehicles and equipment. Pork producers have adopted trailer wash policies where each trailer is washed, disinfected, and dried before it can return to a farm. Cleanliness of livestock trailers after washing is determined by visual inspection rather than any objective method. Adenosine triphosphate (ATP) bioluminescence is used in many industries to provide real-time feedback on surface cleanliness through the detection of ATP from organic sources. That same technology may provide trailer wash facilities a way of objectively characterizing a livestock trailer’s suitability to return to a farm after washing. Two ATP luminometers (3M Clean-Trace and Neogen AccuPoint) were used to estimate the correlation between ATP bioluminescence readings and aerobic bacterial plate counts (APCs) from sampled surfaces and to determine locations within a livestock trailer that can accurately estimate surface cleanliness. Five locations in livestock trailers were evaluated. Those locations included the nose access door (NAD), back door flush gate, rear side access door (RSAD), belly flush gate (BFG), and belly side access door (BSAD). There was a positive log–log association between the two luminometers (*r* = 0.59, *P* < 0.01). Every log unit increase in one unit, resulted in a 0.42 log increase (*P* < 0.01) in the other unit. ATP can come from bacteria, yeasts, molds, and manure. There was a poor association (*r* ≥ 0.10, *P* ≥ 0.02) between APCs and the ATP luminometers. Still, an increase in relative light units (RLUs) resulted in a corresponding increase in colony-forming units. The greatest area of surface contamination measured by APC was the NAD. RLUs were also greater in the NAD compared to the RSAD, the BFG, and the BSAD (*P* ≤ 0.01). Because APCs and luminometer RLUs provided similar outcomes, statistical process control charts were developed to determine control limits for RLUs. This provides real-time feedback to trailer wash workers in determining cleanliness outcomes for livestock trailers. These data suggest that ATP bioluminescence can be a reliable method to monitor cleaning effectiveness in livestock trailers. Bioluminescence is a monitoring tool that should be used in conjunction with microbial methods to monitor procedures for cleaning and disinfection.

## Teaser

Adenosine triphosphate (ATP) bioluminescence technology is used to quantitatively determine the overall cleanliness of surfaces. Greater levels of surface contamination yield a higher relative light units (RLUs) reading. Multiple locations inside livestock trailers were sampled to determine the usefulness of the technology to quickly determine overall cleanliness. ATP luminometers may be used in livestock trailers to determine the general cleanliness of a livestock trailer in tandem with a visual inspection.

## INTRODUCTION

Diseases like Porcine Epidemic Diarrhea virus (PEDv), shown to spread by fomites and carried by contaminated vehicles and equipment, continue to plague the swine industry, costing producers and processors hundreds of millions of dollars annually (Paarlberg, 2014). Because of this, pork producers have adopted commercial trailer wash policies where each trailer is washed, disinfected, and dried before it is allowed to return to a farm. Much like other segments of pork production, costs associated with trailer washing have increased in recent years, partially due to increasing propane prices associated with drying trailers after they are washed. Visual inspection to determine if a trailer is clean usually occurs after the invested cost of propane to dry the truck has occurred. At the same time, studies have demonstrated that visual inspection of cleaned transport trailers may be insufficient to ensure cleanliness and reduce disease transmission risk (Alvarado et al., 2020). However, due to time and laboratory requirements, traditional methods of environmental surveillance like culture, qPCR, and virus isolation are also poor candidates for routine inspection practices. The use of adenosine triphosphate (ATP) bioluminescence has been successfully used to assess equipment cleanliness in production agriculture applications such as in farrowing rooms (Yi et al., 2020) and dairy parlors (Vilar et al., 2008). It is also used outside of production agriculture, including in mixers and food preparation areas (Azizkhan, 2014) and hospitals and medical facilities (Aycicek et al., 2006; Boyce et al., 2009). The objective was to assess the ability of ATP bioluminescence for use as a tool to evaluate livestock trailer cleanliness and disease transmission risk. In doing so, the intention was to enhance wean-to-harvest pig production biosecurity and to improve U.S. swine herd health. The hypothesis was that ATP bioluminescence would provide a rapid, easy-to-use tool capable of monitoring contamination to reduce disease transmission risk associated with livestock transport.

## MATERIALS AND METHODS

No animals were used during this experiment. IACUC approval was not necessary for this experiment.

### Experimental Design

Sampling occurred between April and July 2023 at one of two commercial livestock trailer wash sites. Each location washed approximately 50 trailers every week. One of the facilities was designated to wash trailers that transported high-health pigs (porcine reproductive and respiratory syndrome (PRRS)/PEDv negative farms). This trailer wash location was referred to as the “clean” site. The other trailer wash was designated to wash trailers that transported pigs from locations that were potentially exposed to various infectious diseases (compromised health status sites/PEDv-positive sites). This location was referred to as the “dirty” site. Trailers were initially flushed with fresh water to remove wood chips and manure from the trailer. After the majority of bedding material was removed, trailers were power washed with fresh water to remove any residual material that remained. Following power washing a visual inspection occurred by trailer wash personnel. After the trailer was visually inspected and determined clean, the entire trailer was disinfected using an Accelerated Hydrogen Peroxide (Intervention, Virox Technologies Inc., ON Canada) at a dilution of 1:64.

A target of 50 trailers was sampled from the clean and dirty trailer wash facility (100 trailers total). Every trailer was swabbed in up to five locations using two ATP luminometers to determine variability in ATP readings within a trailer and across luminometers. The 50 trailers sampled at the dirty location were also swabbed for aerobic bacterial plate counts (APCs) to determine total colony-forming units (CFU).

**Figure 1.**
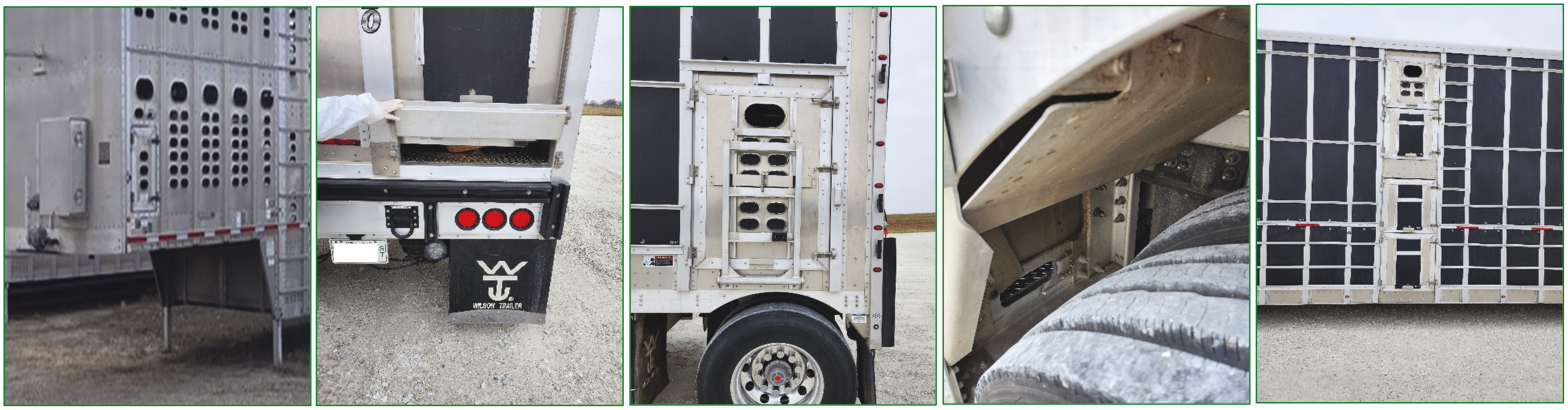
Locations sampled for adenosine triphosphate (ATP) residue using 2 ATP luminometers and bacterial colony-forming units. Trailer locations include from left to right: nose access door (NAD), back door flush gate (BDFG), rear side access door (RSAD), belly flush gate (BFG), and belly side access door (BSAD).

Five locations were identified as being both accessible from the exterior of the trailer and a potential area of contamination and disease transmission due to insufficient cleaning. Those five areas were the nose access door (NAD), back door flush gate (BDFG), rear side access door (RSAD), belly flush gate (BFG), and the belly side access door (BSAD; Fig. 1).

Not every trailer had all five access points, but every available access point was swabbed for every sampled trailer. A total of 409 paired swabs were collected across the five access points, including 15 from the NAD, 94 from the BDFG, 100 from RSAD, 100 from the BFG, and 100 from the BSAD (Table 1).

**Table 1.** Number of observations of ATP bioluminescence swabs by trailer location

Trailer location	ATP luminometer	
	3M Clean-Trace	Neogen AccuPoint
Nose access door (NAD)	15	15
Back door flush gate (BDFG)	94	94
Rear side access door (RSAD)	100	100
Belly flush gate (BFG)	100	100
Belly side access door (BSAD)	100	100
Total	409	409

ATP swabs were collected during a 12-week period as trailers were available for sampling. Swabs were collected from seven trailers in week 1, 9 trailers in week 5, 6 trailers in week 6, 9 trailers in week 7, 7 trailers in week 8, 5 trailers in week 9, 15 trailers in week 10, 17 trailers in week 11, and 25 trailers in week 12 for a total of 100 trailers during the entire sampling period. A total of 208 environmental swabs were collected from livestock trailers at the “dirty” location for culture and molecular testing. Environmental swabs were collected from the same trailers in the same locations as the ATP bioluminescence swabs.

Each trailer was swabbed for environmental bacterial culture to determine total bacterial counts and for real-time reverse transcriptase polymerase chain reaction (RT-rtPCR) to determine the presence of PEDv, porcine deltacoronavirus (PDCoV), and transmissible gastroenteritis virus (TGE) ribonucleic acid (RNA). Those trailers were also included in the ATP swab process to allow for calculating correlations between environmental bacterial swabs, RT-rtPCR for coronavirus detection, and ATP bioluminescence. Swabs intended for viral detection were collected, frozen, and stored at −80 °C until the conclusion of the trial when they were tested by RT-rtPCR.

### Measurement of ATP Bioluminescence

Two individual luminometers and testing kits were used to measure ATP residues on disinfected surfaces. One luminometer was a 3M Clean-Trace luminometer (Neogen Corporation, Lansing, MI). The other was a Neogen AccuPoint luminometer (Neogen Corporation). Each sampler consisted of a clear plastic tube filled with liquid stable luciferin–luciferase reagent and a sterile swab. A 10 cm × 10 cm square was drawn with vertical and horizontal back-and-forth motions to fill the square for each sample for both luminometers. Each square was adjacent or directly next to one another for both luminometers. The AccuPoint sample was swirled for 2 s and then inserted into the clear unibody in the luminometer chamber to measure the relative light unit (RLU) reading. A similar process occurred for the Clean-Trace sample. The Clean-Trace sample was swirled for 5 s and then inserted into the luminometer chamber to measure the RLU reading. The luminometers were calibrated with a positive and negative control between trailers to verify instrument accuracy.

### Aerobic Bacterial Plate Counts

APC on trailer surfaces was estimated by soaking a 10 × 10 cm gauze in 10 mL of Dey/Engly (DE) neutralizing broth (Fisher Scientific, Waltham, MA). Each side was manually pressed on the same area of contaminated surface, and the results were reported as the number of colony-forming units per 100 cm^2^ (CFU/100cm^2^). Exactly 100 µL of D/E neutralizing broth was spread onto standard TSA/sheep blood plates (Fisher Scientific). The plates were incubated for 22 h and the number of colonies per plate was counted. Due to the low surface bacterial loads, all plates were counted with undiluted sample (10°). The total number of colonies per 100 cm^2^ was calculated by multiplying CFU/mL by 10, or the total volume of DE broth used to saturate the sponge. A total of 12 samples were collected from the NAD, 44 from the BDFG, 50 from the RSAD, 49 from the BFG, and 50 from the BSAD.

### Testing for PEDV, PDCoV, and TGE RNA

Viral RNA was extracted from environmental swabs using the MagMAX Core extraction kit on a Kingfisher-96 magnetic particle processor consistent with manufacturer instructions (Applied Biosystems, Foster City, CA). RT-rtPCR was performed using the VetMAX PEDV/TGEV/PDCoV kit. Signal amplification was monitored using a 7500 Fast thermocycler (Applied Biosystems). Cycle thresholds (C_t_s) >36 were considered negative for all three viruses.

### Statistical Analysis

Comparisons of RLUs between luminometers and trailer location were performed by multiple linear regression with log transformations of continuous outcomes before analysis using R statistics software (v 4.3.0). Estimated marginal means were calculated using the emmeans package (v1.8.5). Receiver operating characteristic (ROC) curves and AUC were compared with the pROC package (v1.18.4). Scatterplots and bar graphs were visualized in the ggplot2 package (v3.4.2).

Statistical process control (SPC) critical limits were calculated by first log transforming raw RLU data. Log RLUs were averaged, and a cleanliness threshold was established at +3σ above the mean. Log RLUs can be reverse transformed to report raw RLUs.

Colony-forming units per 100 cm^2^ were first log transformed for normality prior to analysis. Total bacterial counts per surface area were estimated by multiplying the colony-forming counts per milliliter by 10 to get the total estimated bacterial count in the collected sample. The counts were then adjusted by the surface area swabbed with the 10 × 10 cm gauze to estimate viable bacterial colonies per surface area. The final adjustment factor was approximately 10.687. The results were analyzed by analysis of variance using the lm function in R. A small value (0.1) was first applied before log transformation to remove any zero values. A Tukey-Kramer test was applied to all pairwise contrasts between estimated marginal means to control family-wise error rate.

## RESULTS

**Figure 2.**
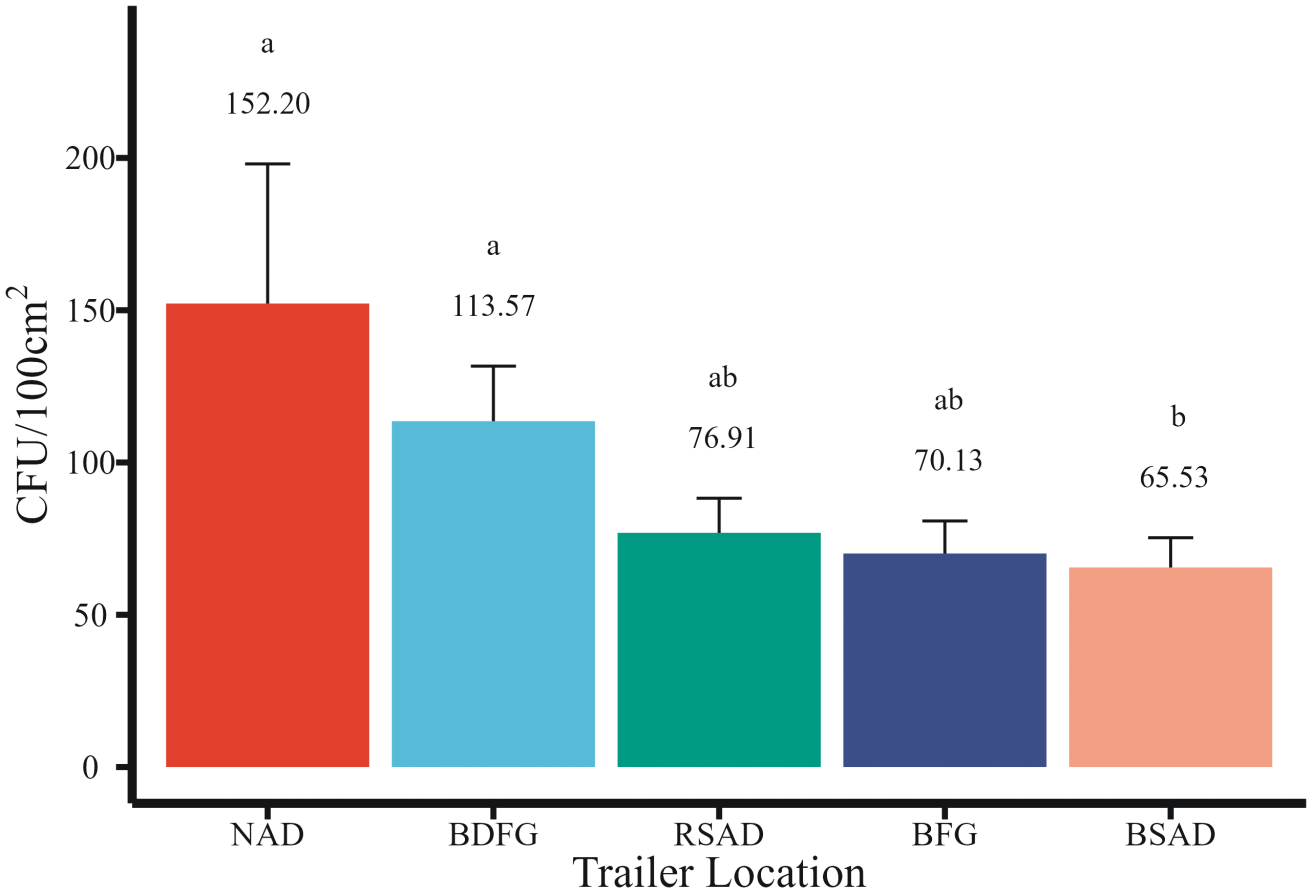
Bacterial counts by trailer location in colony-forming units per 100 cm^2^ (area of 10 cm × 10 cm gauze). Trailer locations include the nose access door (NAD), back door flush gate (BDFG), rear side access door (RSAD), belly flush gate (BFG), and belly side access door (BSAD). Means that do not share a superscript differ (*P* ≤ 0.05).

Bacterial loads were quantified separately at each of the five locations in the trailer (Fig. 2). There were differences of CFU by trailer location (*P* = 0.02). The bacterial load on the NAD was greater compared to the BSAD (ratio 2.32, *P* = 0.10), and BDFG was greater compared to the RSAD (ratio 1.73, *P* = 0.09). There was an overall pattern with the NAD having the greatest bacterial load at 152.20 CFU/100cm^2^ (95% CI: 84.05 to 275.61) followed by the BDFG at 113.57 (95% CI: 83.00 to 155.42), the RSAD at 76.91 CFU/100cm^2^ (95% CI: 57.50 to 102.88), the BFG at 70.13 CFU/100cm^2^ (95% CI: 51.95 to 94.67), and the RSAD at 65.53 CFU/100cm^2^ (95% CI: 48.84 to 87.91). No viral RNA (PEDV, PDCoV, or TGE) was detected in any of the areas sampled, and all had Ct values > 36 (results not shown).

**Figure 3.**
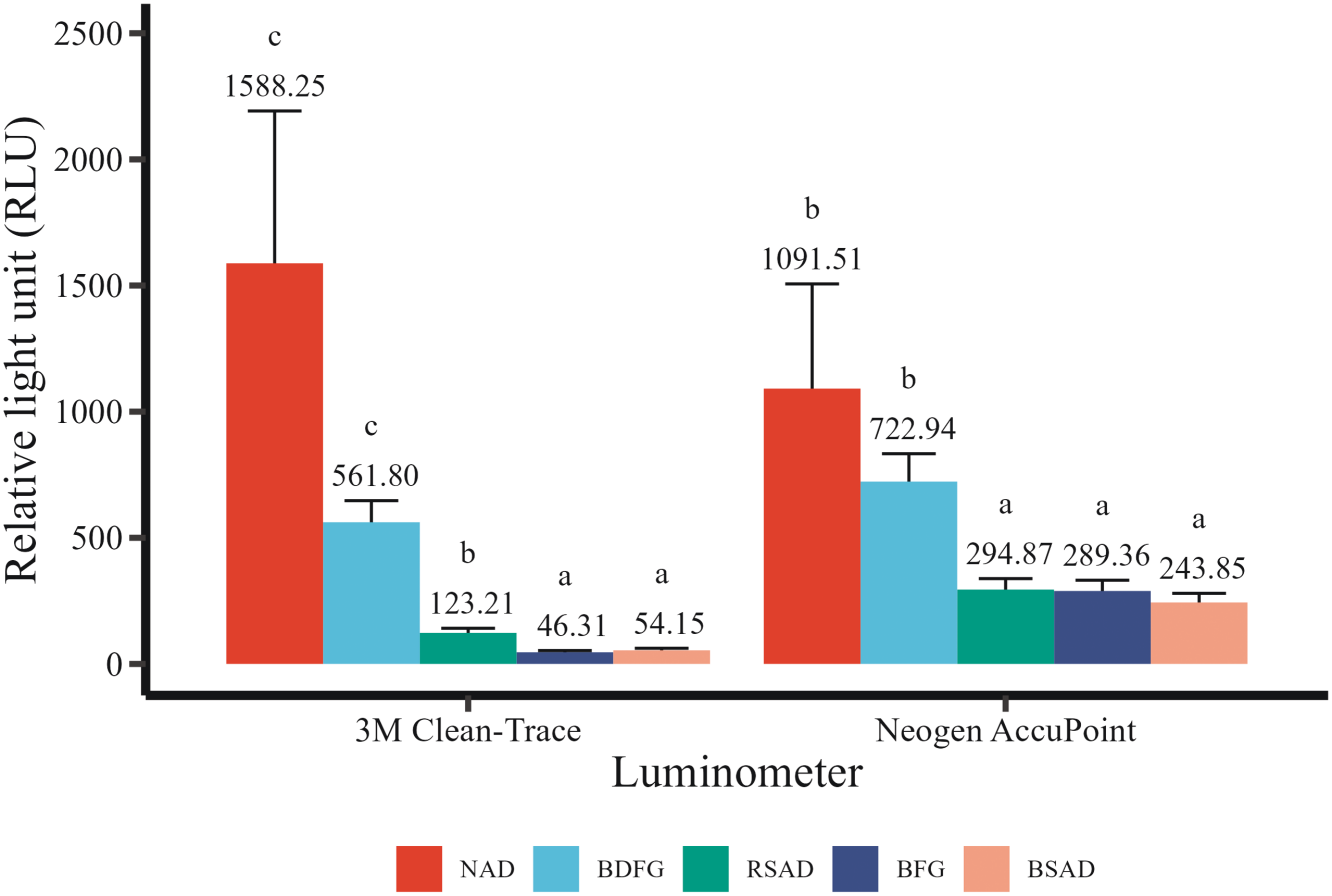
Surface contamination levels across five locations as measured by relative light units (RLU) with two independent ATP luminometers. Trailer locations include the nose access door (NAD), back door flush gate (BDFG), rear side access door (RSAD), belly flush gate (BFG), and belly side access door (BSAD). ATP luminometers were 3M Clean-Trace (Neogen Corporation, Lansing, MI) or Neogen AccuPoint (Neogen Corporation, Lansing, MI). Means within a luminometer that do not share a superscript differ (*P* ≤ 0.05).

The pattern in APC matched closely with RLUs measured with both luminometers (Fig. 3). The greatest RLUs measured by the 3M Clean-Trace luminometer were found in the NAD compared to the RSAD (ratio: 12.80, *P* < 0.01), the BFG (ratio: 34.22, *P* < 0.01), and the RSAD (ratio: 29.28, *P* < 0.01). RLU readings were higher in the NAD compared to the BDFG (ratio: 2.83, *P* = 0.08). The RLUs measured at the BDFG were increased compared to the RSAD (ratio: 4.56, *P* < 0.01), the BFG (ratio: 12.11, *P* < 0.01), and the RSAD (ratio: 10.36, *P* < 0.01). RLU measurements in the RSAD were greater than the BFG (ratio: 2.66, *P* < 0.01) and the BSAD (ratio: 2.27, *P* < 0.01); however, there was no difference between the BFG and the RSAD (ratio 0.86, *P* = 0.94). The Neogen AccuPoint luminometer also reported the greatest RLU readings in the NAD compared to the RSAD (3.70, *P* = 0.01), the BFG (ratio: 3.77, *P* = 0.01), and the BSAD (4.47, *P* < 0.01). RLU readings for the AccuPoint luminometer were greater in the BDFG compared to the RSAD (ratio: 2.45, *P* < 0.01), the BFG (ratio: 2.50, *P* < 0.01), and the BSAD (ratio: 2.96, *P* < 0.01). No differences were detected between the BDFG and the NAD (ratio 0.66, *P* = 0.85) or between any of the RSAD, the BFG, or the BSAD (*P* > 0.89).

**Figure 4.**
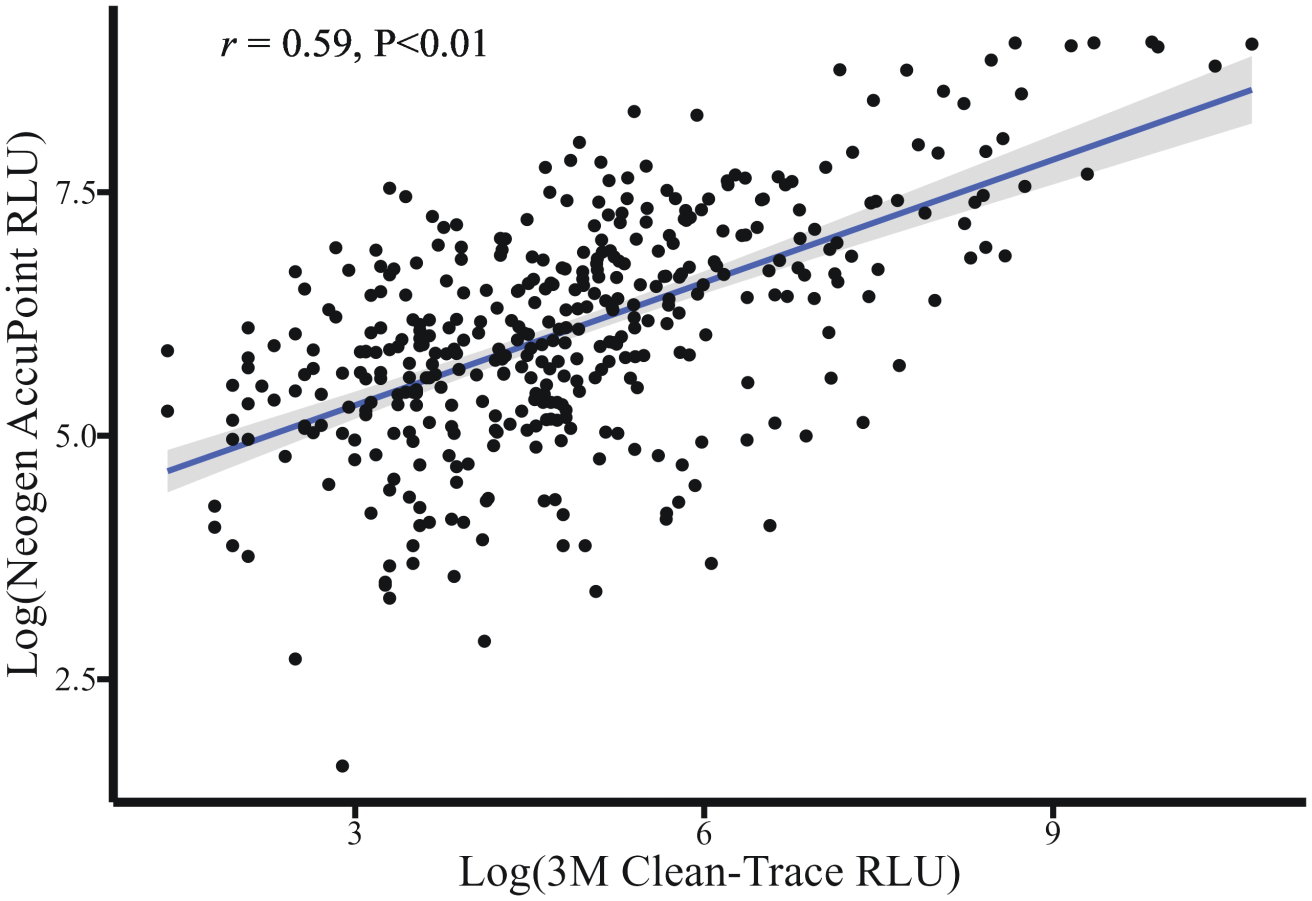
Relationship of relative light units (RLU) between a 3M Clean-Trace luminometer (Neogen Corporation, Lansing, MI) and a Neogen AccuPoint luminometer (Neogen Corporation, Lansing, MI).

**Figure 5.**
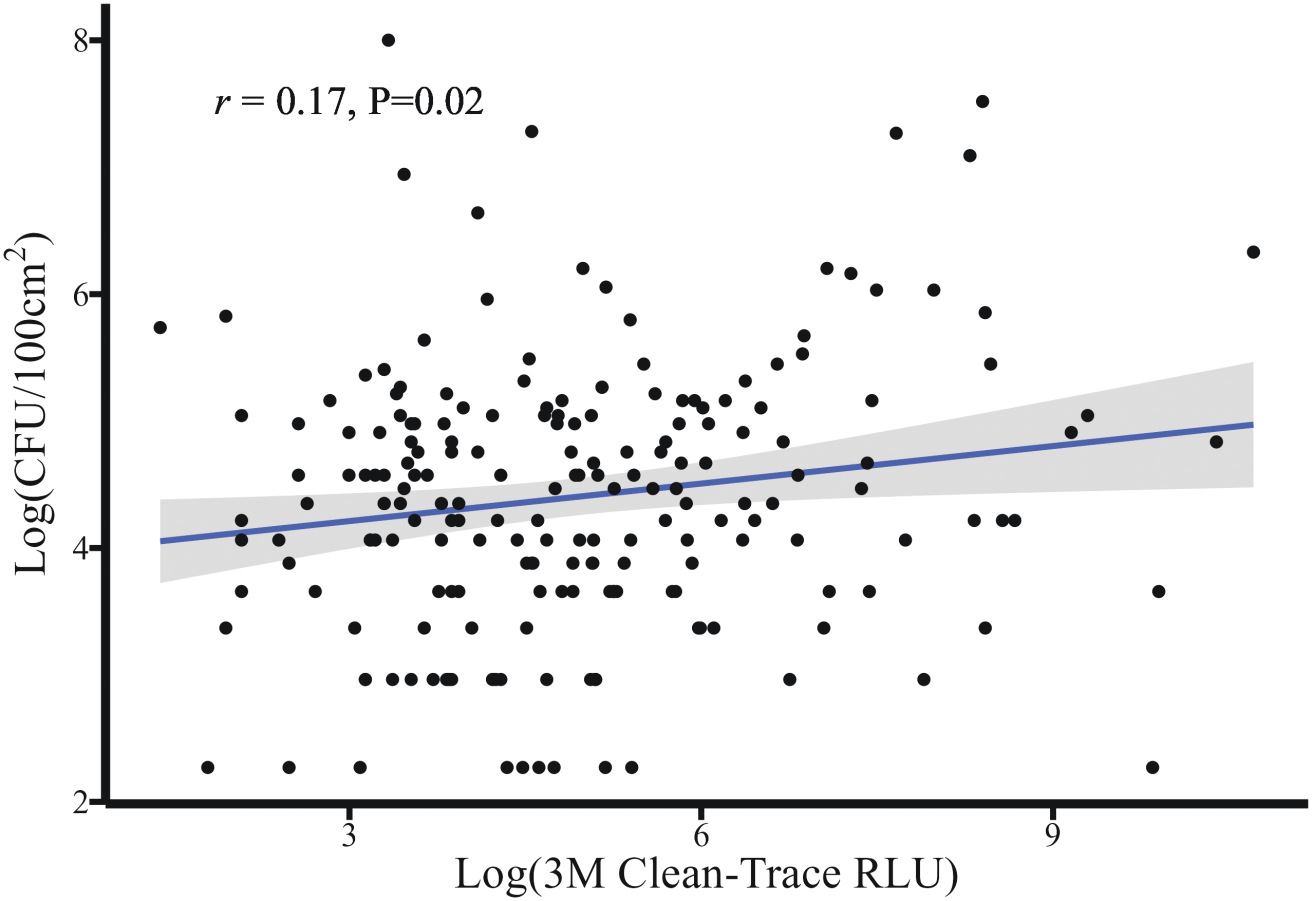
Prediction of colony-forming units (CFU) per 100 cm^2^ of surface area using a 3M Clean-Trace ATP bioluminescence luminometer.

**Figure 6.**
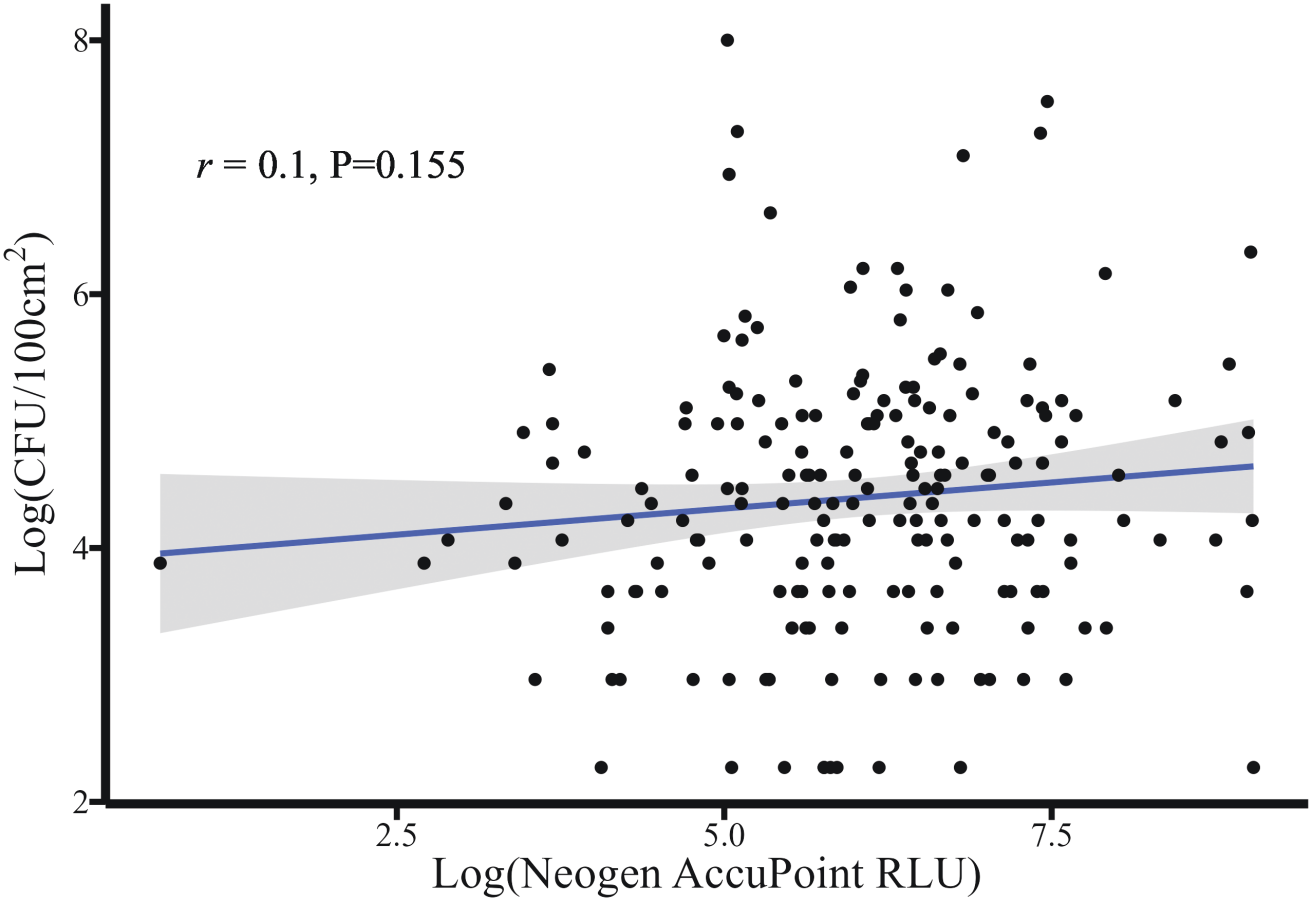
Prediction of colony-forming units (CFU) per 100 cm^2^ of surface area using a Neogen AccuPoint ATP bioluminescence luminometer.

There was a moderate-to-strong positive log–log association (Fig. 4) between the Clean-Trace and the AccuPoint luminometers (*r* = 0.59, *P* < 0.01). For every log unit increase in the Clean-Trace RLU reading, there was a corresponding 0.42 log increase in the RLU read by the AccuPoint luminometer (*P* < 0.01). Overall, there was a poor association between APCs and both the Clean-Trace RLU (*r* = 0.17, *P* = 0.02) and the AccuPoint RLU (*r* = 0.10, *P* = 0.16). The Clean-Trace luminometer (Fig. 5) appeared to correlate with APCs more closely than the AccuPoint luminometer (Fig. 6), with a 0.10 log increase in CFU corresponding to a log unit increase with the Clean-Trace RLU (95% CI 0.02 to 0.18, *P* = 0.02). This association was not different from the AccuPoint RLU readings (slope = 0.08, *P* = 0.16).

ROC curves were developed for each assay using APCs as the gold standard diagnosis of cleanliness. A cutoff of 250 CFU/100cm^2^ was applied from prior studies for the determination of sample status. Samples with CFUs greater than 250 were considered positive. The area under the curve (AUC) was computed by the trapezoidal rule and compared between the two instruments. The AUC for the Clean-Trace luminometer (0.64) was greater than that of the AccuPoint luminometer (0.51, *P* = 0.01). The optimum threshold for the Clean-Trace luminometer was calculated using the Youden’s J statistic, optimizing both sensitivity and specificity. The optimum threshold of 938 resulted in a sensitivity of 50% and specificity of 89%.

## DISCUSSION

ATP bioluminescence is commonly used in healthcare and food processing facilities for assessing cleanliness and surface contamination. However, it has not gained widespread use in the livestock industry. In this study, a total of 409 paired swabs were collected across five locations within commercial swine transport trailers, including 15 from the NAD, 94 from the BDFG, 100 from the RSAD, 100 from the BFG, and 100 from the BSAD. The results from this study indicate the areas of greatest concern in this study were the NAD and the BDFG as detected both by ATP bioluminescence and APC. However, only 15 of the 100 trailers sampled had a NAD access point. Therefore, it is not recommended as a potential monitoring location to determine livestock trailer cleanliness. Instead, the BDFG is the location inside a livestock trailer that had the greatest surface contamination and the most likely available access point. There are many possibilities as to why there were different levels of contamination across different sampling points. It is possible that because NAD is not a common access point on every trailer, it gets overlooked or personnel are not as experienced with how to thoroughly clean this section. Additionally, BDFG was included in more trailers than NAD, but still not every trailer potentially allowing for personnel to overlook this section as well. Of the trailers sampled, RSAD, BFG, and BSAD were represented on every trailer, and the contamination scores of these sections were similar. Differences could also be related to difficulty in cleaning those sections. These data suggest that ATP luminometers can be used to target contaminated areas and could be used in conjunction with APC testing to monitor surface cleanliness and contamination (Figs. 2 and 3).

A moderate-to-strong, positive log–log correlation was estimated between the Clean-Trace and AccuPoint luminometers (Fig. 4). At the individual sample level, the direct correlation between microbial counts and RLUs was moderate to low (Figs. 5 and 6) illustrating the need for environmental bacterial sampling to be used in conjunction with ATP bioluminescence to ensure compliance with cleaning standard operating procedures (SOPs) and overall cleaning effectiveness. It is also important to recognize ATP can originate from multiple sources, such as bacteria, yeasts, molds, and animal waste, and not just those of interest. These findings are consistent with previous published results, supporting the conclusion that ATP bioluminescence may be used to determine the real-time cleanliness of various surfaces (Larson et al., 2003; Willis et al., 2007; Alvarado et al., 2020). Although the correlations were low between surface CFUs and ATP RLUs, the relationships were consistent with reviews from medical literature where 78.5% (11 of 14) of studies reviewed reported a significant correlation between ATP bioluminescence and other microbiological methods (Nante et al., 2017). The Clean-Trace, AccuPoint, and microbial APC data taken together suggest the luminometers are measuring true surface cleanliness rather than nonspecific background contamination from surface residue not related to the cleaning process (e.g., swab contamination during the sampling process). These residues may include sources such as rinse water, residual bedding, organic material, birds, and human contamination. All of them can impact RLU readings and impact the correlations between ATP and CFU. Even though great care was taken to ensure that the sampled surface areas were immediately adjacent to each other, cleanliness can vary widely across sampled surfaces, increasing variability between measurements.

**Figure 7.**
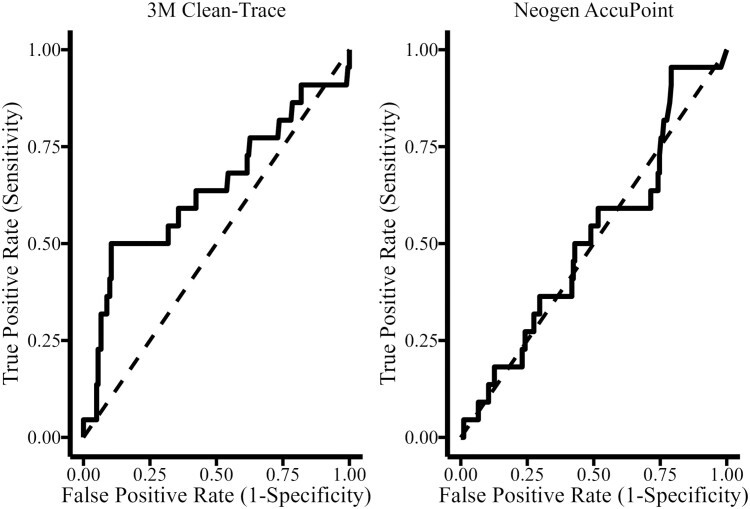
Comparison of performance between 3M Clean-Trace and Neogen AccuPoint luminometers by comparing the area under the receiver operating characteristic curve (ROC) for each instrument. Aerobic plate counts as the gold standard of cleanliness for comparison with 250 colony-forming units (CFU) per cm^2^ as the threshold.

The primary objective of ATP testing was to verify overall cleaning effectiveness, not just to serve as a surrogate for APC testing. Still, the performance between the two luminometers were compared to predict positive aerobic plate counts. A threshold level of 250 CFU/100 cm^2^ was selected as a pass–fail threshold based on prior studies, and the AUC for the two luminometers was compared (Ching et al., 2021). An AUC of 0.50 indicates that a classifier lacks predictive ability and is no different than random chance, while AUCs approaching 1.00 indicate perfect classification. While the performance of the Clean-Trace (AUC 0.63) luminometer was improved to that of AccuPoint (AUC 0.51), neither accurately predicted APCs (Fig. 7).

In general, it is important to consider ATP bioluminescence is a rapid method to verify cleaning procedures. Microbial tests provide outcomes to verify sanitation status. Together they provide a more complete assessment of the effectiveness of the cleaning and sanitation process. ATP is a biological marker that is intended to assess direct (microbial) and indirect (organic matter) hazards that may result in contamination or transmission of infectious material. Detection of organic matter is critical, as it is an indication of effectiveness of the “cleaning process” but also may indicate “growth niches” where organic matter resides, microbial proliferation can occur or viruses can become critical fomites.

The performance of bioluminescence-based ATP test as a hygiene indicator depends largely on the surveillance purpose. These tests tend to perform better in areas that are prone to carrying high microbial loads, as the relative load of ATP contributed by microbial cells is increased. High surface ATP detected when CFU counts were low could be due to organic residue left in the absence of surface microbial contamination.

## CONCLUSION

The use of ATP bioluminescence for rapid feedback of surface cleanliness of livestock trailers is promising. The usefulness of the technology will need to be easy to implement and easy to interpret for full adoption. Therefore, SPC was evaluated as a means to manage risk associated with failure to achieve cleanliness. It is important to note that the adoption of ATP bioluminescence will require the determination of critical limits based on the luminometer of choice and baseline surface cleanliness. Critical limits should be monitored for changes in cleaning procedure, effectiveness, and calibration of equipment.

**Figure 8.**
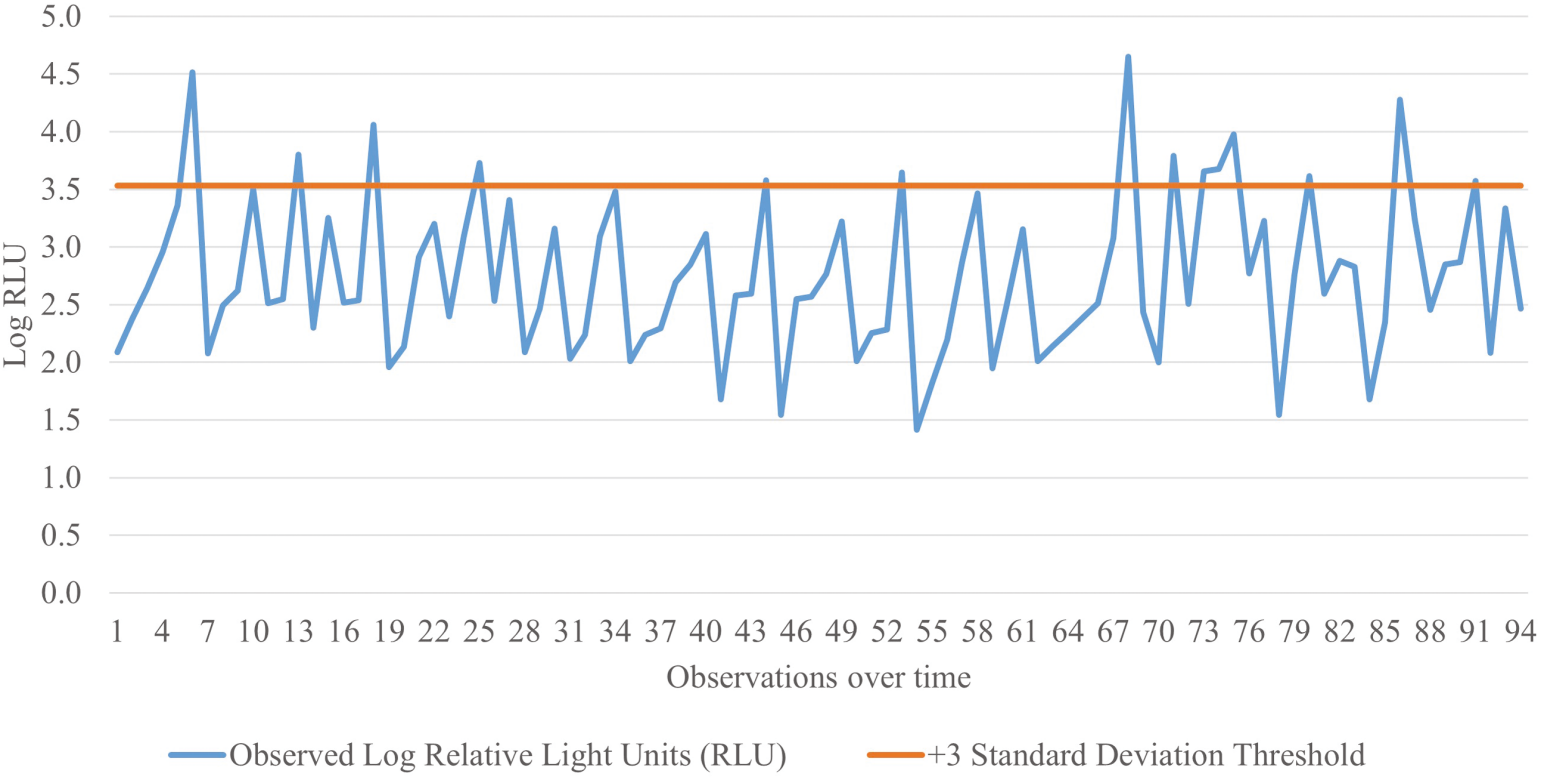
Illustrative example of a statistical process control (SPC) chart depicting log relative light units (RLU) from the back door flush gate (BDFG) using a 3M Clean-Trace ATP luminometer. The average log RLU at this location was 2.7 log. A cleanliness threshold was established as + 3σ above the mean. The cleanliness threshold for this trailer location using this luminometer would be 3.5 log (3,404 reported RLUs). Swabs exceeding this threshold would fail the cleanliness evaluation and require corrective action.

These data support the use of monitoring the back door flush gate with a 3M Clean-Trace luminometer. However, specific critical limits should be determined for each unique situation prior to implementation. Using a 3 SD threshold for failure, 14% (14 of 100) of the livestock trailers evaluated in this experiment would have failed the cleanliness threshold (Fig. 8). Using these data to generate SPC charts provides real-time feedback to trailer wash personnel on the effectiveness of the wash and thereby reduces the risk of disease transmission when the trailer returns to a farm. It is possible that false positives or negatives may occur. However, the cost of another wash may be less expensive than an infectious disease outbreak such as PEDv or PRRSV. ATP bioluminescence provides a tool that aids in risk management. Critical limits may be adjusted to be more or less (increase or decrease the number SDs from the mean) restrictive based on producers’ tolerance for risk. Bioluminescence is a monitoring tool that should be used in conjunction with microbial methods to monitor procedures for cleaning and disinfection.
